# Maternal near miss and mortality attributable to hypertensive disorders in a tertiary hospital, Tanzania; a cross-sectional study

**DOI:** 10.1186/s12884-020-02930-y

**Published:** 2020-05-18

**Authors:** Jane R. Manyahi, Hans Mgaya, Ali Said

**Affiliations:** Department of Obstetrics and Gynaecology, Muhimbili University of Health and Allied Sciences, P.O. Box 65001, Dar es Salaam, Tanzania

**Keywords:** Maternal near-miss, Maternal death, Severe pre-eclampsia, Eclampsia, Tanzania

## Abstract

**Background:**

Hypertensive disorders in pregnancy is the second most common direct cause of maternal deaths accounting for 14% of maternal deaths worldwide. Severe pre-eclampsia and eclampsia are among the hypertensive disorders in pregnancy causing significant morbidity and mortality, hence categorized as Maternal Near Miss. At Muhimbili National Hospital these are the leading causes of maternal deaths accounting for 19.9% of maternal death. This study aimed to determine the proportion of severe maternal outcomes and maternal near-miss indices among patients with severe pre-eclampsia and eclampsia at Muhimbili National Hospital in Tanzania.

**Methods:**

A descriptive cross-sectional study was conducted between September 2017 to January 2018 at Muhimbili National Hospital. Women with severe pre-eclampsia and eclampsia were recruited. Data were extracted from patient files after admission, and followed up until discharge or death; after discharge was categorized as maternal near miss or death as maternal death. The outcome indicators were calculated using the total number of live births during the study period, the number of maternal deaths and maternal near-miss due to severe pre-eclampsia/ eclampsia in the same period.

**Results:**

Nearly two-thirds of women recruited, 199 (62.2%) had severe preeclampsia while 121 (37.8%) had eclampsia, 71 (22.1%) had severe maternal outcome whereby 63 had maternal near-miss with organ dysfunction and 8 maternal deaths. The overall maternal near-miss ratio was 87.4 while that for severe pre-eclampsia was 54, and 33 per 1000 live births for eclampsia. Overall severe maternal outcome ratio was 19.4 while that for severe pre-eclampsia was 12 and that for eclampsia was 9.5 per 1000 live births. Mortality index was 11% and the Case fatality rate was 2.5%.

**Conclusion:**

There is a high proportion of women with severe maternal outcome attributable to severe pre-eclampsia and eclampsia, with a reduced proportion of maternal deaths. This signifies improvement of performance in our facility in dealing with patients with severe morbidities due to severe pre-eclampsia and eclampsia, however, more effort should be put to further reduce maternal mortality**.**

## Background

Hypertensive disorders in pregnancy are the second most common direct cause of maternal death accounting for 14.0% of maternal deaths Worldwide, 12.9% in high- income countries, 14% in low- income countries [1]. In Tanzania, it is among the commonest direct causes of maternal deaths. At Muhimbili National Hospital (MNH) the major causes are pre-eclampsia and eclampsia which accounts for 19.9% of maternal deaths [2].. Tanzania has shown progress towards improving maternal health, as is shown by a 47% decline in maternal mortality ratio (MMR) from an estimated MMR of 870 per 100,000 live births in 1990 to an MMR of 460 per 100,000 live births in 2010 [3]. The progress, however, has not been consistent, as in 2015–16 MMR was 556 per 100,000 [4]. All the same, this indicates that there is a gap in the provision of obstetric and maternal care which needs to be addressed.

Previously, the evaluation of maternal health care services aimed at improving the quality of obstetric care traditionally relied on enquiries into maternal deaths. Review of cases with severe maternal morbidity, described as “near miss” those who nearly died, is a useful complement to the investigation of maternal mortality [5]. World Health Organization (WHO) identifies some severe maternal morbidities to be in MNM criteria which are the potentially life-threatening conditions which include: postpartum haemorrhage, severe pre-eclampsia, eclampsia, sepsis or severe systemic infection, ruptured uterus, and severe complications of abortions, critical intervention or intensive care unit admission and organ dysfunction. In hypertensive disorders, severe pre-eclampsia and eclampsia, intensive care unit admission and organ dysfunction are categorized as a maternal near miss [6].

They proposed the following indicators for the evaluation of care in women with severe maternal morbidities. These indicators are severe maternal outcome ratio (SMOR) and the maternal near-miss ratios (MNMR), which are outcome indicators that provide an assessment of how frequently those conditions occurred in the source population, hence help to provide an estimate of the complexity of care that is required by the population served by the health-care facility. Higher ratios indicate that a substantial proportion of cases will require more complex interventions to survive their complications. Other indicators are mortality index (MI) and the maternal near-miss mortality ratios (MNM: 1MD) these provide an estimate of performance. These help to asses the quality of care provided to these patients, higher ratios indicate that the quality of care provided to severe cases may need to be reviewed [6].

A near miss approach is an important tool in the evaluation and assessment of the newer strategies for improving maternal health worldwide. Applying t WHO MNM approach Worldwide will help to facilitate comparisons between different studies, and between countries and regions, to have uniformity of reporting and analyzing severe maternal morbidity events, hence address shortcomings in the quality of obstetric care and improve pregnancy outcome [6]. This report will give a snapshot to the World to what extent hypertensive disorders in pregnancy can lead to maternal near-miss and maternal death in our institution. Comparison with other countries or settings can be made by applying WHO maternal near-miss approach, hence intervention can be made to reduce maternal near-miss and maternal attributable to Hypertensive disorders in pregnancy Worldwide. Moreover, little information was available on the severe maternal outcome due to severe pre-eclampsia and eclampsia which are among the leading causes of severe maternal outcome Worldwide. This study aims to determine the proportion of severe maternal outcome and MNM indices among patients with severe pre-eclampsia and eclampsia at MNH by applying the WHO MNM approach.

## Methods

### Study design and settings

A descriptive cross-sectional study was conducted between September 2017 to January 2018 at MNH, which is the largest public and tertiary hospital in Tanzania and teaching hospital for the Muhimbili University of Health and Allied Sciences. It caters for a population of about 4.4 million Dar es Salaam residents. The hospital receives patients from 3 Regional hospitals and other hospitals within the city and upcountry. Being a tertiary hospital it receives patients with severe morbidities that need specialized care.

Maternity block has six antenatal and postnatal wards, which can accommodate 40 patients each. Critically ill patients due to hypertensive disorders and other obstetric conditions are admitted in ward 35. This ward has two units, one is an ICU unit where patients who need intensive care including mechanical ventilation are admitted. The other unit is a high dependency unit for patients with life-threatening conditions including severe pre-eclampsia and eclampsia who will not need mechanical ventilation. An average of 20 patients with severe pre-eclampsia and eclampsia are admitted per week. All basics investigations are done to all patients admitted in ward 35, other investigations are done as needed. Magnesium sulphate is initiated to all patients with severe pre-eclampsia and eclampsia, to prevent/ treat convulsion. Antihypertensives are used to control blood pressure. The on-call team (admitting team) on each day includes one specialist obstetrician/ gynaecologist, two residents, and one intern doctor, they are responsible for daily morning and evening ward rounds. There is also a team of intensivists and anesthesiologists who are responsible for intubation of patients who need mechanical ventilation, daily reviewing and discharging patients from ICU. There is also a multi Profesional consultation system where specialists from other departments are called to review patients who need their attention. Patients who need dialysis are sent to the dialysis unit according to their schedules.

#### Outcome measures

The primary outcome measures were the total number of MD and MNM due to severe pre-eclampsia/ eclampsia during the study period. The total number of live birth during the study period were obtained and used to calculate maternal near-miss indices.

Subsequently outcome indicators were calculated as proposed by WHO near-miss approach such as: Case-specific maternal near-miss ratio due to pre-eclampsia and eclampsia, Case-specific severe maternal outcome ratio due to severe pre-eclampsia/ eclampsia, Case fatality rate due to severe pre-eclampsia/ eclampsia, Case-specific mortality index due to severe pre-eclampsia/eclampsia, and Case-specific maternal mortality near-miss ratio due to severe pre-eclampsia/eclampsia.

#### Inclusion criteria

We included all patients with severe pre-eclampsia: these were patients who presented with Systolic blood pressure of 160 mmHg or higher, or diastolic blood pressure of 110 mmHg, or with features of organ dysfunction, and eclampsia these were patients who presented with generalised fits in a woman without a previous history of epilepsy. Include coma in pre-eclampsia and maternal death according to WHO definition [6]. The severe maternal outcome was considered fulfilled if patients with severe pre-eclampsia /eclampsia presented with any organ dysfunction as per laboratory criteria, or clinical criteria, or critical intervention plus maternal death. The definitions of criteria, the applicability in our setting and how we interpreted them are presented in Table A, Additional file 1.

#### Data collection

Convenient sampling technique was used whereby; all women with severe pre-eclampsia and eclampsia were identified and recruited on a daily basis by the principal researcher through daily review of admission books in ward 35 and admitting wards. Where inclusion criteria were met, the researcher recruited the patients and followed them during hospitalization until their discharge or death.

A structured data abstraction form was filled which included: patients particulars such as age, parity, gravidity, gestation age, referral status, mode of delivery, hypertensive disorder: severe pre-eclampsia or eclampsia. Critical intervention: admission to ICU and maternal outcome organ dysfunction and maternal death. This data was obtained from patients files, in case of missing information or doubt the facility medical staff was questioned.

Organ dysfunction was diagnosed using laboratory results which were obtained from the patient’s file or computer database. Once women were discharged, they were considered to have survived and constituted a near-miss case, those who died constituted maternal death. Any maternal death due to severe preeclampsia and/or eclampsia, which occurred before being recruited in the study was also included. A woman with severe pre-eclampsia/eclampsia who presented with a least one of the organ dysfunction was considered as MNM with organ dysfunction, together with maternal death they constituted SMO.

#### Data analysis

The collected data were coded, entered, cleaned and analyzed using the Statistical Package for the Social Sciences (SPSS), Version 22. Categorical variables were summarized as frequencies and percentage. Continuous variables were summarized into mean and standard deviation. The proportion of Severe maternal outcome among patients with severe Preeclampsia and eclampsia were obtained by summation of all maternal deaths due to severe pre-eclampsia/eclampsia and patients with severe pre-eclampsia/eclampsia with any organ dysfunction**.**

The outcome indicators were calculated using the total number of Live Births (LB) during the study period, the number of maternal deaths and maternal near-miss due to severe pre-eclampsia/ eclampsia in the same period. These indicators were as follows;

Case-specific maternal near-miss ratio due to pre-eclampsia and eclampsia were calculated by the number of maternal near-miss cases due to pre-eclampsia and eclampsia per 1000 live births. Case-specific severe maternal outcome ratio due to severe pre-eclampsia/ eclampsia determined as the number of women with life-threatening conditions(organ dysfunction)plus maternal death due to severe pre-eclampsia/eclampsia (MNM + MD) per 1000 live birth. Case fatality rate due to severe pre-eclampsia/ eclampsia was determined as the proportion of deaths out of the total number of patients presenting with severe pre-eclampsia and eclampsia, expressed as a percentage. Case-specific mortality index due to severe pre-eclampsia/eclampsia was determined as the number of maternal deaths due to severe pre-eclampsia and eclampsia divided by the number of women with life-threatening conditions due to severe pre-eclampsia/ eclampsia expressed as a percentage. Case-specific maternal mortality near-miss ratio due to severe pre-eclampsia/eclampsia (MNM: 1 MD) determined as the ratio between maternal near-miss cases due to severe pre-eclampsia/ eclampsia and one maternal death due to severe pre-eclampsia /eclampsia.

#### Ethical clearance and consideration

The ethical clearance for the study was obtained from the Senate Research and Publication Committee of Muhimbili University of Health and Allied Sciences (reference number MU/PGS/SAEC/Vol.X/59) on 19th July 2017. Permission to conduct the study was granted from the Executive Director of Muhimbili National Hospital where the study was conducted. Data was collected and extracted from the patient’s records without identification of the subject. A data abstraction form was filled using the patient’s records and therefore inclusion did not interfere with the management of the patient. Considering the precautions above, individually obtained informed consent was not required. Due to the fact above infromed consent was waived by the Senate Research and Publication Committee of Muhimbili University of Health and Allied Sciences. Data were treated confidentially, the patient was identifying by folder number and filled form was kept on a locked cabinet only accessible to the principal investigator. During follow up of the patients, the principal investigator worked together with admitting team by consulting them regarding treatment and participated in some of the procedures to make sure these patients received necessary care and treatment.

## Results

During the study period, there were 3853 deliveries, whereby 3661were live births. A total of 320 women were recruited in this study, whereby 310 (96.9%) women were referred from peripheral hospitals. They presented with severe pre-eclampsia and eclampsia, 199 (62.2%) and 121 (37.8%) respectively. All patients with severe Preeclampsia and eclampsia, 320 (100%) received Magnesium Sulphate as per hospital protocol**.** Mean age was 27.1 ± 6.31 years, more than two-thirds 247 (77.2%) were aged 19–35 years. A high proportion of women were para 0 and para1–2 with 136(42.5%) and 129(40.3%) respectively. Mean gestational age at delivery was 34.0 ± 3.5 weeks; the majority of them had premature delivery at GA of 28–36 weeks 225 (70.3%). Out of 320 pregnant women, 27 (8.4%) were admitted to ICU.

## Discussion

Severe pre-eclampsia and eclampsia are hypertensive disorders in pregnancy which cause a significant proportion of maternal morbidity and mortality in Tanzania. Index study is one of the studies describing maternal morbidity and mortality due to severe pre-eclampsia and eclampsia by applying WHO MNM approach. The high proportion of SMO among patients with severe pre-eclampsia and eclampsia in our study lies within the wide range of ratios reported in other studies from other low-income countries [7, 8].

In the index study, MNMR found to be higher compared to other studies done in low and middle-income countries. This indicates that there are a high proportion of women who presented with severe morbidities due to hypertensive disorders, hence required more care and resources to survive the complications. This result was comparable to the previous study done in the same setting [9]. A low MNMR was however reported in different studies done in low and middle-income countries [7, 10, 11]. The difference could probably be because in our study more than 90% of women were referred from peripheral facilities with morbidities already, compared to 20 to 64%in the above studies. It could also be due to the difference of quality of care for hypertensive disease in pregnancy in different settings, as they had a high proportion of MD as compared to MNM. The care in our facility, being a tertiary facility, is likely to be more appropriate than that offered in peripheral facilities, hence explaining the high proportion of MNM cases coming from these facilities but they survived.

In the current study, severe maternal outcome ratio was19.4 per 1000 live births, whereby severe preeclampsia accounted for higher ratio than eclampsia.WHO recommends SMOR below 10 per 1000 live births. High SMOR in this study indicates that a substantial proportion of women required more complex intervention to survive their complications. Despite the interventions some women died from their complications, this shows that more effort has to be put to save the lives of these patients with severe maternal morbidities. Ninety per cent of these participants were referred from peripheral health facilities or home already with complications, this explains the high SMOR. The results are comparable with the findings from the previous study conducted in the same setting [9]. Other studies were done in low-income settings, however, reported low SMOR [7].

Mortality index (MI) provides an estimate of how a health facility is performing in dealing with complex and severe cases. WHO recommends the mortality index of below 5%. (6)In this study it was found to be high; mortality index of 11%. This was similar to what other authours in low-income settings found [7, 8]. A high mortality index was however reported in different studies in low-income settings [10, 12]. This demonstrates that a high proportion of critically ill women in low-income settings die from their complications. The mortality index found in this study is higher than WHO recommendations but low when you compare with the findings of other studies in low-income settings. Having a well organized and functioning obstetric ICU and high dependency unit could explain on the improvement of performance in caring of critically ill patients, but more effort needs to be put to reduce maternal deaths to the recommended standards.

In this study, the overall case-fatality 2.5% was high, whereby eclampsia accounted for higher ratio than severe preeclampsia. WHO recommends Case fatality rate among women with direct obstetric complications in emergency health facilities to be less than 1% [13]. This was low, comparable to previous studies done in the same setting in 2006 and 2009 [14, 15] and other studies were done in different low-income countries [7, 16, 17]. This implies that a substantial proportion of patients with severe morbidities survived the complication, hence reduction of maternal deaths among these patients. Attention is needed on eclampsia as it still has a high case fatality rate and mortality index, to reduce maternal death to acceptable standards.

Maternal Near Miss Mortality ratio is the ratio of MNM per one maternal death: a higher ratio indicates better care. In this study, it was found that in every forty women with severe pre-eclampsia/eclampsia one woman died. This implies that a high proportion of patients with severe maternal morbidity survived these complications as compared to the previous study done in the same setting [9], but still unacceptable ratio as per WHO guideline. This indicates improvement in the care provided to these patients in our facility as explained above. These findings are contrary to a previous study done in other African countries which had a low ratio of around 20:1 [7, 16, 17].

The strength of this study is that women were recruited after admission and followed up to the day of discharge or death. This enabled the principal investigator to have up to date data. The limitation of this study was that there were no reagents for renal function tests for 2 weeks during the study period. Blood samples of women with the clinical presentation of acute kidney injury were sent to Muhimbili Orthopedic Institute (MOI) for the tests. Hence some women with no symptoms of acute kidney injury missed these investigations**.** We adapted WHO MNM criteria but due to limited resources, some laboratory investigations such as blood PH and lactate, and management based criteria such as interventional radiology were not used, however, this had no impact on diagnosis and outcome since other clinical-based criteria were applied (Tables 1, 2 and 3).

**Table 1:** Distribution of maternal characteristics among women with severe pre-eclampsia and eclampsia at MNH (*N* = 320)

Maternal Characteristics	Frequency (%)
**Age group**	
< 19	38 (11.9)
19–35	247 (77.2)
more 35	35 (10.9)
**Parity**	
0	136 (42.5)
1–2	129 (40.3)
3–4	47 (14.7)
≥ 5	8 (2.5)
**Gravidity**	
1	117 (36.6)
2–4	171 (53.4)
≥ 5	32(10.0)
**Gestational age at delivery**	
< 28 weeks	15 (4.7)
28–36 weeks	225 (70.3)
≥ 37 weeks	80 (25)
*Mode of delivery	
Vaginal delivery	157 (49.4)
Caesarian section	161 (50.6)
**Admission to ICU**	
Yes	27(8.4)
No	293(91.6)
**Referral status**	
Yes	310(96.9)
No	10(3.1)
**Hypertensive disorders**	
Severe pre-eclampsia	199(62.2)
Eclampsia	121(37.8)

*n **= 318 Two (0.6%) women died undelivered**

**SEVERE MATERNAL OUTCOME**

Haemolysis Elevated Liver enzymes Low Platelet count (HELLP) syndrome was the leading dysfunction whereby 58(18.1%) of participants presented with it. Out of 320 women, 71 women presented with SMO (63 MNM with organ dysfunction and 8 maternal deaths), whereby 56.3% had severe preeclampsia while 43.7% had eclampsia. Severe preeclampsia had a high proportion of organ dysfunction of 57.1%. Eclampsia had a high proportion of MD as compared to severe preeclampsia. **(**Table 2**)**

**Table 2:** Distribution of severe maternal outcomes among women with severe pre-eclampsia and eclampsia at MNH (*N* = 320)

Maternal outcomes	Severe pre-eclampsia	Eclampsia	Total
Placenta abruption			
Yes	12(70.6)	5(29.4)	17
No	187(61.7)	116(38.3)	303
Maternal death			
Yes	4(50.0)	4(50.0)	8
No	195(62.5)	117(37.5)	312
Cardiovascular dysfunction			
Yes	4(66.7)	2(33.3)	6
No	195(62.1)	119(37.9)	314
Respiratory dysfunction			
Yes	5(55.6)	4(44.4)	9
No	194(62.4)	117(37.6)	311
Renal dysfunction			
Yes	9(42.9)	12(57.1)	21
No	190(63.5)	109(36.5)	299
HELLP syndrome			
Yes	34(58.6)	24(41.1)	58
No	165(63.0)	97(37.0)	262
Neurological dysfunction			
Yes	0(0.0)	8(100.0)	8
No	199(63.8)	113(36.2)	312
*Organ dysfunction			
Yes	36(57.1)	27(42.9)	63
No	163(63.4)	94(36.6)	257
SMO			
Yes	40(56.3)	31(43.7)	71
No	159(63.9)	90(36.1)	249

*Organ dysfunction = total number of women with organ dysfunction

**Table 3:** Maternal morbidity and mortality indicators among women with severe pre-eclampsia and eclampsia at MNH

Indicator	Indices calculations	Overall ratio	Hypertensive Disorder	Case specific ratio
Case specific maternal near miss ratio (MNMR)	MNM/LBX1000	87.4	S. Preeclampsia	54
(per 1000 LB)			Eclampsia	33
Case specific severe maternal outcome ratio(SMOR) (per 1000 LB)	(MNM + MD)/LBX1000	19.4	S. Preeclampsia	12
			Eclampsia	9.5
Mortality index (MI) (as percentage)	MD/(MNM + MD)X100	11	S. Preeclampsia	9
			Eclampsia	11.4
Case fatality rate (CF) (as the percentage)	MD/Total number of pts. with severe pre-eclampsia and eclampsia X 100	2.5	S. Preeclampsia	2.1
			Eclampsia	3.3
Case specific maternal near miss mortality ratio	MNM:1MD	40:1	S. Preeclampsia	50:1
			Eclampsia	30:1

***LB*Live Birth**

The overall maternal near-miss ratio was 87.4. The overall case-fatality rate was 2.5% whereby severe preeclampsia accounted for 2.1% while eclampsia accounted for 3.3% respectively. **(**Table 3**)**

## Conclusion

In conclusion, our study found a high proportion of women with severe maternal outcome among patients with severe pre-eclampsia and eclampsia, with a reduced proportion of maternal deaths. This signifies improvement of performance in our health facility in dealing with patients with severe morbidities due to severe pre-eclampsia and eclampsia. More effort, however, should be put to further reduce maternal mortality. There is a need for future interaction with primary health facilities, so as interventions can be made from lower health in order to reduce severe maternal morbidities and mortality. Moreover, the results could be used to develop policies and/or protocols for improving health care for women at every level.

## Supplementary information

**Additional file 1: **Table A WHO near miss criteria adapted according to Hypertensive disorders in pregnancy and local **c**ontext of Muhimbili National Hospital.

## Data Availability

The data sets generated and analysed during the current study are not publicly available due to the issue of confidentiality but are available from the corresponding author on reasonable request.

## Abbreviations

MNM: Maternal Near Miss
WHO: World Health Organization
HDP: Hypertensive Disorders in Pregnancy
SMO: Severe Maternal Outcome
MI: Mortality Index
SMOR: Severe Maternal Outcome Ratio
MNMR: Maternal Near Miss Ratio
MD: Maternal Death, LB-Live Births
