# Supplementary material for: Maternal near miss and mortality attributable to hypertensive disorders in a tertiary hospital, Tanzania; a cross-sectional study
Source: BMC Pregnancy Childbirth. 2020 May 18;20:301. doi: 10.1186/s12884-020-02930-y (PMC7236332; doi:10.1186/s12884-020-02930-y)
Supplement: Supplementary file 1 — Additional file 1: Table A WHO near miss criteria adapted according to Hypertensive disorders in pregnancy and local context of Muhimbili National Hospital. [file 12884_2020_2930_MOESM1_ESM.docx]

**Additional File 1:**

**Table A:**

**WHO near miss criteria adapted according to HDP and local context of MNH**

| **WHO near miss criteria** | **MNH criteria in HDP and local context** |
| --- | --- |
| 1. **Severe maternal complications** |  |
| - Severe Pre eclampsia | - Severe Pre eclampsia |
| - Eclampsia | - Eclampsia |
| - Severe postpartum haemorrhage |  |
| - Sepsis or severe systemic infection |  |
| - Ruptured uterus |  |
| **B.Critical interventions or intensive care unit use** |  |
| - Admission to intensive care unit | • Admission to intensive care unit among patient with HDP |
| - ***Interventional radiology** |  |
| - Laparotomy (includes hysterectomy, excludes caesarean section |  |
| - Use of blood products |  |
| **C. Life-threatening conditions (organ dsyfunction)** |  |
| - *****Cardiovascular dysfunction   Shock, cardiac arrest (absence of pulse/ heart beat and loss of consciousness), use of continuous vasoactive drugs, cardiopul- monary resuscitation, **severe hypoperfusion (lactate >5 mmol/l or >45 mg/dl), severe aci- dosis (pH <7.1)** | - Cardiovascular dysfunction   Shock, cardiac arrest (absence of pulse/ heart beat and loss of consciousness), use of continuous vasoactive drugs, cardiopul- monary resuscitation in patients with HDP |
| - Renal dysfunction   Oliguria non-responsive to fluids or diuretics, dialysis for acute renal failure, severe acute azotemia (creatinine ≥300 µmol/ml or ≥3.5 mg/dl) | - Renal dysfunction   Oliguria non-responsive to fluids or diuretics, dialysis for acute renal failure, severe acute azotemia (creatinine ≥300 µmol/ml or ≥3.5 mg/dl) in patients with HPD |
| - Coagulation/haematological dysfunction   Failure to form clots, massive transfu- sion of blood or red cells (≥5 units), severe acute thrombocytopenia (<50 000 platelets/ml) | - Coagulation/haematological dysfunction   Failure to form clots, massive transfu- sion of blood or red cells (≥5 units), severe acute thrombocytopenia (<50 000 platelets/ml) in patients with HPD |
| - Hepatic dysfunction   – Jaundice in the presence of pre eclampsia, severe acute hyperbilirubinemia (bilirubin >100 µmol/l or >6.0 mg/dl), ASAT and ALAT to twice normal | - Hepatic dysfunction   Jaundice in the presence of pre eclampsia, severe acute hyperbilirubinemia (bilirubin >100 µmol/l or >6.0 mg/dl), ASAT and ALAT to twice normal in patients with HPD |
| - Neurological dysfunction   Prolonged unconsciousness (lasting ≥12 hours)/coma (including metabolic coma), stroke, uncontrollable fits/status epilepticus, total paralysis | - Neurological dysfunction   Prolonged unconsciousness (lasting ≥12 hours)/coma (including metabolic coma), stroke, uncontrollable fits/status epilepticus, total paralysis |
| - Uterine dysfunction - Uterine haemorrhage or infection leading to hysterectomy |  |
| **D.Maternal vital status**:   - Maternal death: is the death of a woman while pregnant or within 42 days of termination of pregnancy irrespective of duration and site of pregnancy from any cause related to or aggravated by the pregnancy or its management, but not from accidental or incidental causes | **Maternal vital status**:   - Maternal death: Death during pregnancy or within 42 days of termination of pregnancy irrespective of duration and site of pregnancy in patients with severe pre eclampsia/eclampsia |

**Note** .

**The study was conducted before the current classification of hypertensive disorders, the 2019 ACOG Practice Bulletin no 202.Hence the classification used was the former one.**

**WHO SMO refers to life threatening condition (organ dysfunction), including all maternal near miss case and maternal death .In our paper SMO refers to patients with severe pre eclampsia/ eclampsia with any organ dysfunction plus maternal death.**

*** A= Admission to Intensive care unit was the only inclusion criteria applied among management based criteria , patients with severe pre eclampsia and eclampsia who underwent laparotomy and used blood products were included in the study. Interventional radiology is not done in our institution due to limited resources**

***B= Cardiovascular dysfunction- due to limited resources laboratory investigations such as blood PH and lactate are not done in our institution.**
